# Supplementary material for: Antioxidant and Polyphenol-Rich Ethanolic Extract of Rubia tinctorum L. Prevents Urolithiasis in an Ethylene Glycol Experimental Model in Rats
Source: Molecules. 2021 Feb 14;26(4):1005. doi: 10.3390/molecules26041005 (PMC7917717; doi:10.3390/molecules26041005)
Supplement: Supplementary file 1 [file molecules-26-01005-s001.pdf]

molecules

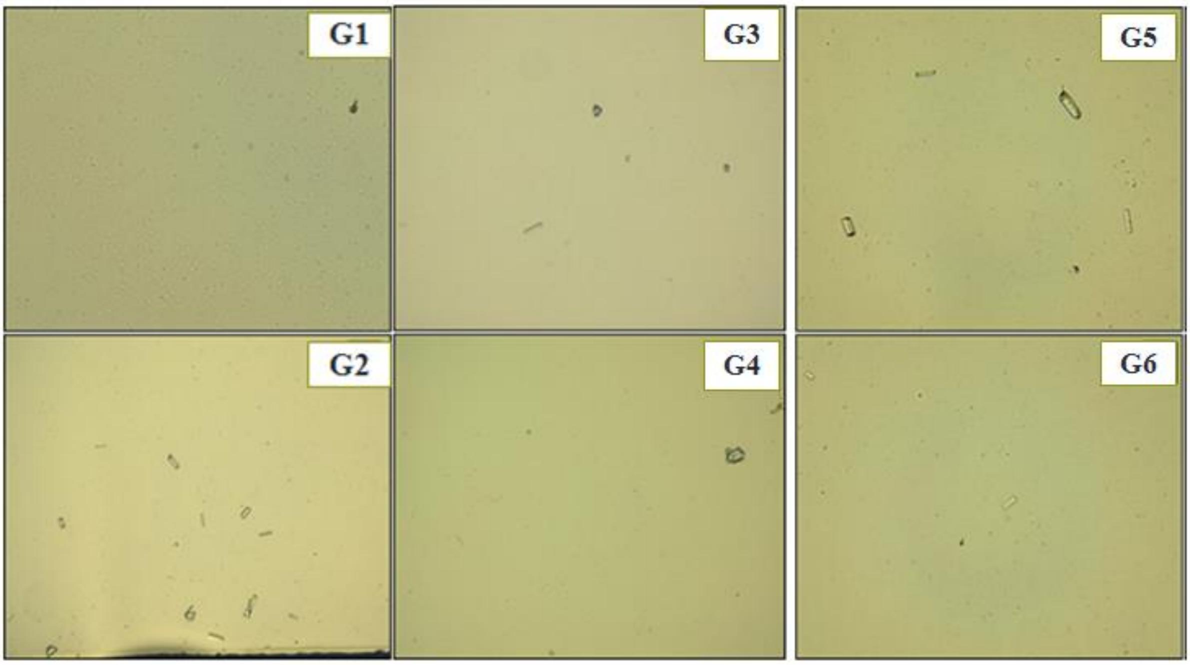

Figure S1. Typical calcium oxalate (CaOx) crystals, viewed under light microscope (50×), in 24h urine from rats of vehicle control (**G1**), lithiasic group (**G2**), groups treated with E-RT at 1 g/kg (**G3**) and 2 g/kg (**G4**) and groups treated with EA-RT at 1 g/kg (**G5**) and 2 g/kg (**G6**).

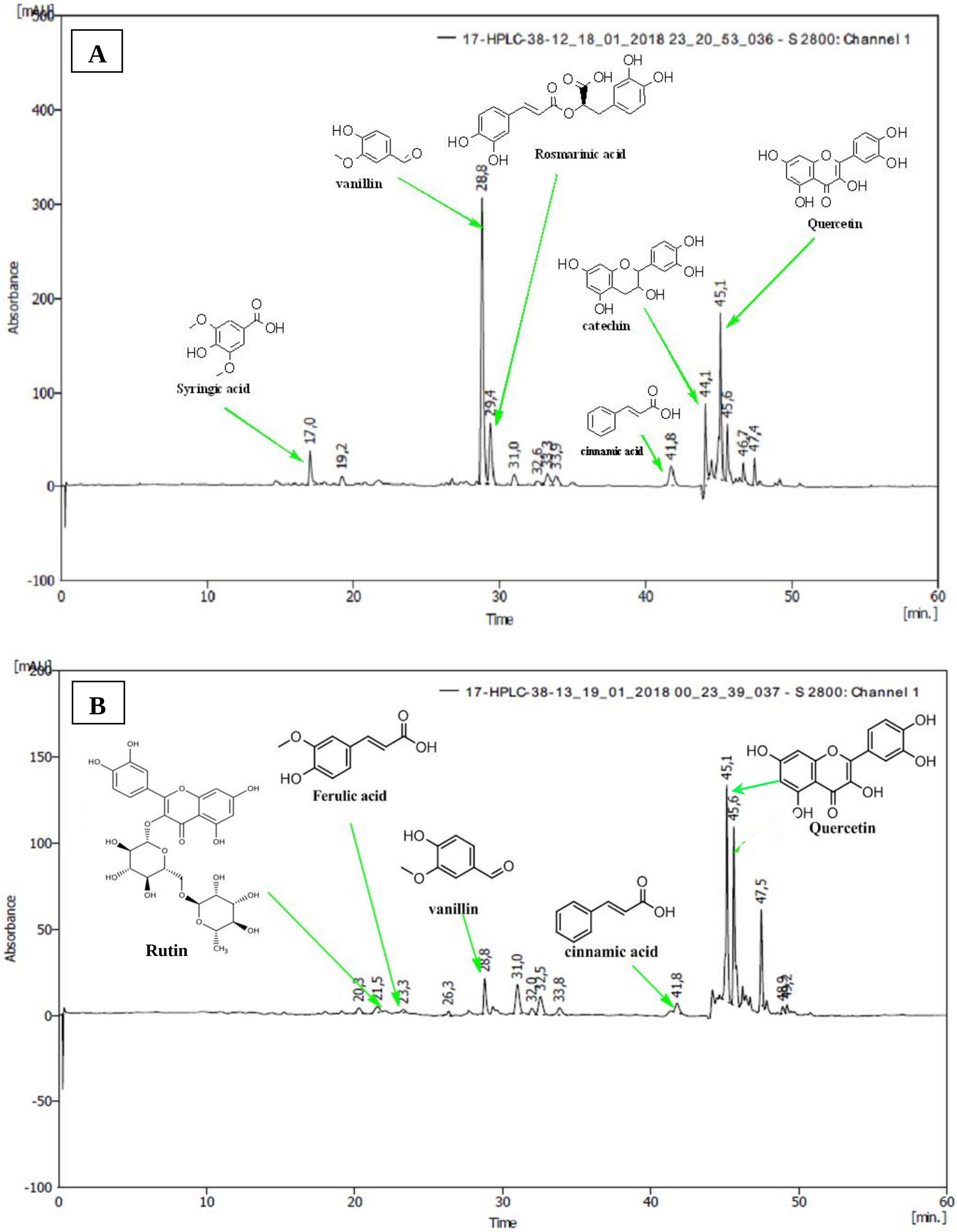

Figure S2. *HPLC* chromatograms of E-RT **(A)** and EA-RT **(B).** Syringic Acid (R_t =17.0),), rutin (R_t =21.5), ferulic Acid (R_t =23.3), vanillin (R_t =28.8), rosmarinic acid (R_t =29.4), cinnamic acid (R_t =41.8), catechin (R_t =44.1), quercetin (R_t =45.1).
